# Programmed death-ligand 1 and tumor-infiltrating lymphocytes (TILs) – low TIL density may predict poorer long-term prognosis in T1 laryngeal cancer

**DOI:** 10.1007/s00428-023-03586-7

**Published:** 2023-07-18

**Authors:** Pihla Pakkanen, Taru Ilmarinen, Elina Halme, Heikki Irjala, Petri Koivunen, Matti Pukkila, Sami Ventelä, Alhadi Almangush, Eva-Maria Birkman, Outi Lindgren, Virva Pohjolainen, Nelli Sjöblom, Caj Haglund, Jaana Hagström, Leena-Maija Aaltonen

**Affiliations:** 1 Department of Otorhinolaryngology - Head and Neck Surgery, University of Helsinki and Helsinki University Hospital, Helsinki, Finland; 2 Department of Otorhinolaryngology - Head and Neck Surgery, University of Tampere and Tampere University Hospital, Tampere, Finland; 3 Department of Otorhinolaryngology - Head and Neck Surgery, University of Turku and Turku University Hospital, Turku, Finland; 4 Department of Otorhinolaryngology - Head and Neck Surgery, University of Oulu and Oulu University Hospital, Oulu, Finland; 5 Department of Otorhinolaryngology - Head and Neck Surgery, University of Eastern Finland and Kuopio University Hospital, Kuopio, Finland; 6 Department of Pathology, University of Helsinki and Helsinki University Hospital, Helsinki, Finland; 7 Department of Pathology, University of Turku and Turku University Hospital, Turku, Finland; 8 Department of Pathology, University of Oulu and Oulu University Hospital, Oulu, Finland; 9 Fimlab Laboratories, Department of Pathology, Tampere University Hospital, Tampere, Finland; 10 Department of Surgery, University of Helsinki and Helsinki University Hospital, Helsinki, Finland; 11 Department of Oral Pathology and Radiology, University of Turku, Turku, Finland

**Keywords:** Early laryngeal cancer, T1 glottic cancer, Tumor-infiltrating lymphocytes, Programmed death-ligand 1, Prognosis

## Abstract

We evaluated the prognostic role of programmed death-ligand 1 (PD-L1) and tumor-infiltrating lymphocytes (TILs) in T1 glottic laryngeal squamous cell carcinoma (LSCC). T1 glottic LSCC patients (*n* = 174) treated at five Finnish university hospitals between 2003 and 2013 were included. Tissue microarray (TMA) blocks were used for PD-L1 immunohistochemistry. TILs were scored from intratumoral and stromal regions in whole tissue sections. Of 174 patients, 92 (53%) had negative, 66 (38%) intermediate, and 16 (9%) high PD-L1 levels. Of 80 patients whose TILs were analyzed, 50 (63%) had low and 30 (38%) high stromal TIL density. Patients with a local recurrence or a new primary tumor of the larynx had lower TIL density than had other patients (*p* = 0.047). High PD-L1 expression with low stromal TIL density was associated with inferior 5-year disease-specific survival (85% vs. 100%, *p* = 0.02). In conclusion, in patients treated for T1 glottic LSCC, low stromal TIL density was associated with local recurrences and new primary tumors of the larynx. High PD-L1 expression with low stromal TIL density may be associated with worse survival in T1 glottic LSCC.

## Introduction

The prognosis of T1 glottic laryngeal squamous cell carcinoma (LSCC) is excellent. Nevertheless, over 10% of patients develop a local recurrence regardless of treatment method [1]. Early detection of LSCC recurrence is significant since curative treatment is frequently possible. A biomarker or a histological feature predicting recurrences of LSCC could be helpful to develop more individualized follow-up protocols.

Programmed death-ligand 1 (PD-L1) is a transmembrane protein whose overexpression could lead to T cell autoinhibition and down-regulation of immune responses. PD-L1’s receptor programmed death protein 1 (PD-1) is an immune checkpoint protein expressed on the surface of immune cells [2, 3]. In head and neck squamous cell carcinoma (HNSCC) the prognostic role of PD-L1 is contradictory [4–6]. However, high PD-L1 expression in tumor and/or immune cells may predict better local control, overall survival (OS) and disease-free survival in stage I-IV LSCC [7–10]. Additionally, high PD-L1 expression in tumor and immune cells is associated with a lower recurrence rate after postoperative radiotherapy in LSCC [11]. Two PD-1/PD-L1 inhibitors, nivolumab and pembrolizumab, have been approved for the treatment of advanced or recurrent HNSCC, and positive PD-L1 expression in tumor and/or immune cells is predictive for treatment response [5, 12–14].

Tumor-infiltrating lymphocytes (TILs) play an important role in the tumor microenvironment of HNSCC [15]. TILs are immune cells which have migrated to the tumor tissue and participate in immune responses during cancer progression [16]. High TIL density may predict better local control, disease-specific survival (DSS) and OS in stage I-IV LSCC [7, 9, 17, 18]. Elevated levels of TILs are furthermore related to improved DSS and recurrence-free survival (RFS) in patients with recurrent or persistent LSCC [19, 20]. The results are similar in other HNSCCs [16]. An established cutoff point to stratify high and low TIL density is not yet validated in HNSCC.

The association between PD-L1 and TIL levels in HNSCC is controversial. For example, in oral squamous cell carcinoma PD-L1 overexpression is associated with both high and low TIL rates. Low TIL rates presenting with high PD-L1 levels suggest that the PD-1/PD-L1 pathway may inhibit T cell activation. Conversely, PD-L1 overexpression with high TIL density can predict better clinical outcome with stronger local immune responses. However, used clones and cutoff points vary between the studies [5, 6].

Published studies on HNSCC often include heterogeneous patient populations with tumors from different anatomical sites and different stages. The aims of the study were to compose a national comprehensive series of T1 glottic LSCC samples from all Finnish university hospital biobanks and to investigate the prognostic role of PD-L1 and TILs.

## Materials and methods

### Patients

Medical records and surgical pathology reports from all patients with T1 glottic LSCC, treated at the five Finnish university hospitals (Helsinki, Turku, Tampere, Oulu, and Kuopio) between 2003 and 2013 were recorded. We included 174 patients with archived paraffin-embedded tissue blocks available for tissue microarray (TMA). The samples were collected from the regional biobanks of all university hospitals. LSCC tumors diagnosed more than 5 years after the primary T1 glottic LSCC tumor were defined as second primary tumors.

The study was approved by The Ethics Committee of Surgery in the Hospital District of Helsinki and Uusimaa and the institutional study permission was granted. According to Finnish law, informed consent from the patients was not needed due to the retrospective study design.

### Tissue blocks

Tissue microarray (TMA) blocks were prepared from primary tumors by each university hospital biobank. The study pathologist reviewed hematoxylin and eosin (H&E) stained slides, and representative 1–2 mm cores from each tumor were detached and placed in a paraffin block. The slides of TMA blocks were stained using PD-L1 antibodies. Whole sections were used for TIL analysis. In the previous studies, both TMA blocks and whole tissue slides have been used for PD-L1 analysis [4].

### Programmed death-ligand 1

In all 174 specimens, immunohistochemistry was performed using PD-L1 antibody (clone SP142, Ventana Medical Systems, Inc., Tucson, Arizona, USA, 7 ug/ml dilution). One TMA block was stained with another PD-L1 clone 22C3 (IHC PharmDx, Dako, Carpinteria, CA, USA) which is currently used in clinical diagnostics in our clinic. The control TMA slide included 25 of 174 (14%) patient samples, and PD-L1 expression was identical with both PD-L1 clones. Combined positive score (CPS) was calculated. CPS is the number of PD-L1 positive cells (tumor cells, lymphocytes, macrophages) divided by the total number of tumor cells, multiplied by 100. In tumor cells, membranous immunostaining was interpreted as positivity. CPS under 1 was graded as negative, 1–20 as low, and 21–100 as high. Tumor proportion score (TPS) was reported to demonstrate how PD-L1 is expressed in tumor cells. TPS is the number of PD-L1 positive tumor cells divided by the total number of tumor cells, multiplied by 100%. No positivity was graded as negative, 1–50% as low, and 51–100% as high. Two researchers (J.H., P.P.), blinded to clinical data, scored the samples.

### Tumor-infiltrating lymphocytes

A subset of 80 tissue blocks from Helsinki University Hospital was further processed for TIL analyses. HE stained slides were used and TILs were scored from both intratumoral and stromal regions. The density of TILs was evaluated as the percentage of the area occupied by infiltrating lymphocytes. TILs were analyzed using the protocol of International Immuno-Oncology Biomarkers Working Group [21].

We tested several different cutoff points for high and low stromal and intratumoral TILs. The best prognostic value for stromal TILs was at 10% (low 0–10%, high 11–100%) and for intratumoral TILs 1% (low 0–1%, high 2–100%). Two researchers (P.P., A.A.) scored all slides and the third researcher (J.H.) analyzed the cases with discrepancy.

### Statistical analyses

For the statistical analyses, we used IBM SPSS Statistics 27.0 (Armonk, NY, USA). Chi-square and Fisher’s tests were used to study a connection between categorical variables and t-test and Mann–Whitney-U test were used for continuous variables. Local control was defined as the duration from diagnosis to the first documented local recurrence. Overall survival (OS) was defined as the duration from diagnosis to death from any cause and disease-specific survival (DSS) to the death caused by LSCC. Survival was analyzed by Kaplan–Meier method and log-rank test. A statistically significant p value was set at 0.05.

## Results

### Clinical characteristics

The clinicopathological characteristics of study patients are presented in Table 1. In total, 174 tumors were analyzed for PD-L1 and 80 for TIL. The median age of all patients at diagnosis was 67 years (range 29–88 years). All patients were treated by curative intent, either with surgery (*n* = 100, 58%) or with radiotherapy (*n* = 74, 42%). Transoral endoscopic surgery was used for the majority of patients and only 1 patient was treated with open surgery. Radiotherapy was external beam radiotherapy and a typical dose was 66 Gy in 2-Gy fractions. Primary treatment method or smoking did not associate with 5-year OS, local control or DSS.

**Table 1:** Clinical characteristics, and expression of PD-L1 and TILs in patients with T1 glottic LSCC (N=174)

Characteristic	All patients (N=174), n (%)	PD-L1 (n=174), n (negative, low, high)	Stromal TILs (n=80), n (low, high)
Sex			
Male	146 (84)	146 (64, 56, 26)	70 (45, 25)
Female	28 (16)	28 (11, 11, 6)	10 (5, 5)
Smoking history			
Yes	147 (84)	147 (58, 59, 30)	70 (43, 27)
No	19 (11)	19 (12, 6, 1)	7 (6, 1)
NA	8 (5)	8 (5, 2, 1)	3 (1, 2)
Earlier dysplasia*			
Yes	12 (7)	12 (5, 5, 2)	6 (4, 2)
No	143 (82)	143 (62, 55, 26)	68 (44, 24)
NA	19 (11)	19 (8, 7, 4)	6 (2, 4)
Histological grade			
Grade 1	50 (29)	50 (23, 15, 12)	14 (11, 3)
Grade 2	51 (29)	51 (21, 24, 6)	23 (16, 7)
Grade 3	5 (3)	5 (2, 2, 1)	3 (2, 1)
NA	68 (40)	68 (29, 26, 13)	40 (21, 19)
T stage			
T1a	156 (90)	156 (64, 62, 30)	73 (45, 28)
T1b	18 (10)	18 (11, 5, 2)	7 (5, 2)
Primary treatment			
Surgery	100 (57)	100 (45, 34, 21)	53 (30, 23)
Radiotherapy	74 (43)	74 (30, 33, 11)	27 (20, 7)
Local recurrence			
Yes	20 (11)	20 (8, 7, 5)	6 (6, 0)
No	154 (89)	154 (67, 60, 27)	74 (44, 30)
Died of LSCC			
Yes	4 (2)	4 (0, 3, 1)	3 (3, 0)
No	170 (98)	170 (75, 64, 31)	77 (47, 30)

Abbreviations: LSCC, laryngeal squamous cell carcinoma; NA, not available; PD-L1, programmed death-ligand 1; TIL, tumor-infiltrating lymphocyte

*presence of dysplasia confirmed in laryngeal biopsy specimens before the diagnosis of T1 glottic cancer

Of all patients 163 (93%) had a minimum 3-year follow-up or until death. In some patients, follow-up continued in smaller hospitals in collaboration with university hospitals. Of 174 patients, 20 (12%) developed a local recurrence in 5-year follow-up, and 1 (0.6%) developed a distant recurrence. Larynx preservation rate was 94% (*n* = 164). Four of 174 (2%) died of LSCC in 5-year follow-up. Of 174 patients, 20 (11%) developed a second primary tumor: 8 in lungs, 7 in larynx, and 5 in other head and neck areas.

### Programmed death-ligand 1

**Fig. 1:**
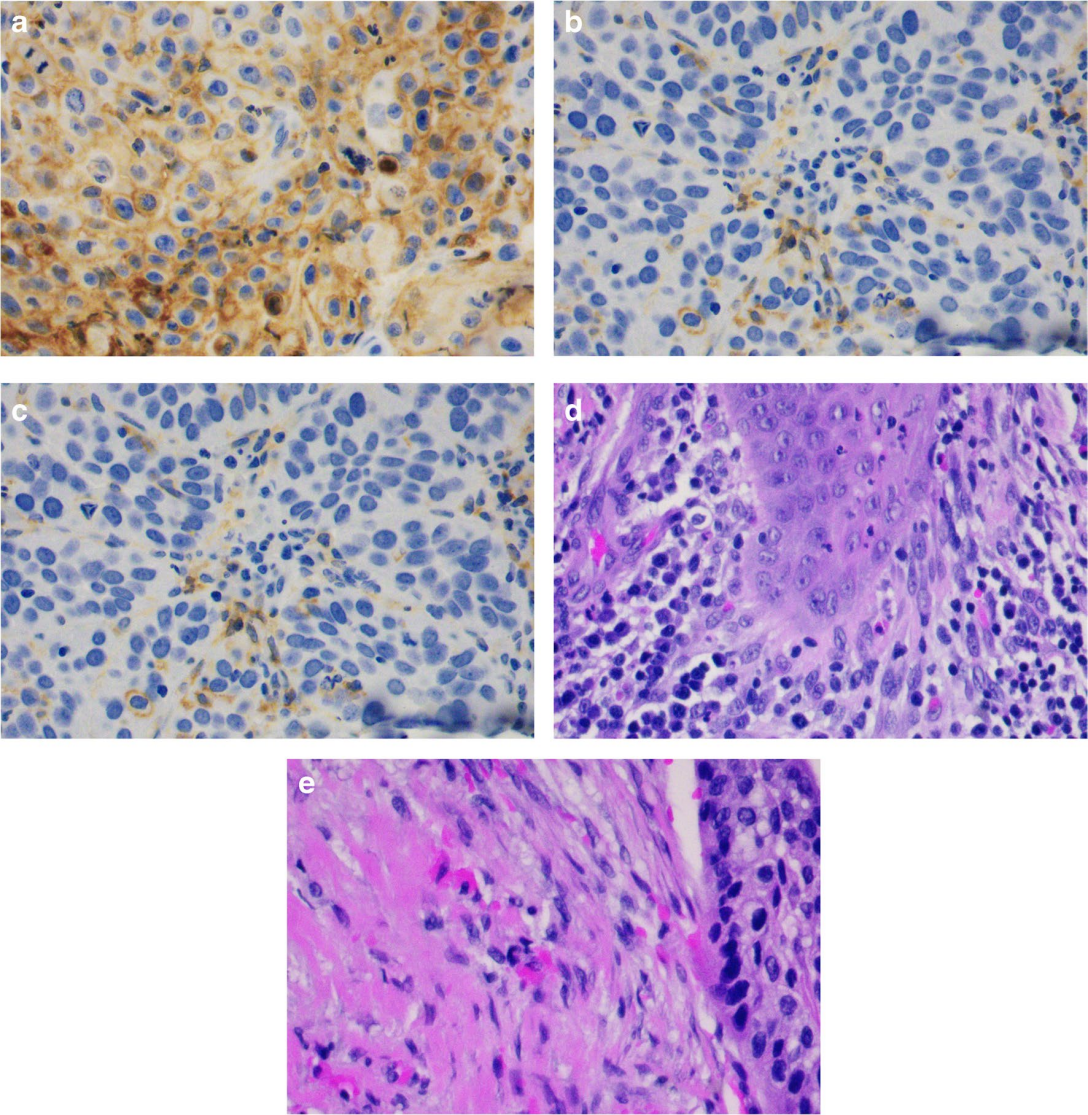
Representative images of immunohistochemistry, magnification × 40. **A**) High PD-L1 (21–100). **B**) Low PD-L1 (1–20). **C**) Negative PD-L1 (< 1). D) High stromal TILs (11–100%). E) Low stromal TILs (0–10%)

Of 174 patients included in PD-L1 analyses, 75 (43%) had negative, 67 (39%) low, and 32 (18%) high PD-L1 levels (Fig. 1). PD-L1 expression was not correlated with sex, smoking, earlier dysplasia, T stage or primary treatment method. PD-L1 was not associated with larynx preservation rate, 5-year OS, local control, or DSS in a log-rank test. When the patients treated with surgery and radiotherapy were analyzed separately, PD-L1 was not associated with local control, DSS, or OS. All patients who died of LSCC had positive PD-L1 status (CPS ≥ 1), but the difference was not statistically significant. There were no differences in previous variables when PD-L1 negative patients were compared to PD-L1 positive patients (CPS < 1 vs. CPS ≥ 1). The correlation between CPS and TPS is presented in Table 2. Of 24 patients presenting with negative TPS, 23 had low CPS, and 1 had high CPS. Thus, in these 24 patients, PD-L1 only presented in the lymphocytes. Of 75 patients presenting with negative CPS, 7 had low TPS. In these patients, PD-L1 only presented in tumor cells.

**Table 2:** The correlation of combined positive score (CPS) and tumor proportion score (TPS) in T1 glottic cancer

N = 174	TPS negative (*n* = 92)	TPS low (*n* = 66)	TPS high (*n* = 16)
CPS negative (*n* = 75)	68	7	0
CPS low (*n* = 67)	23	41	3
CPS high (*n* = 32)	1	18	13

*CPS*, combined positive score; *TPS*, tumor proportion score

### Tumor-infiltrating lymphocytes

Of 80 patients, 30 (38%) had high stromal TIL density, and 50 (63%) low stromal TIL density. High intratumoral TIL percentage group included 11 (14%) patients and low intratumoral TIL percentage group 69 (86%) patients. Stromal and intratumoral TIL percentages were not associated with sex, age, smoking history, earlier dysplasia, T stage, or primary treatment method.

**Fig. 2:**
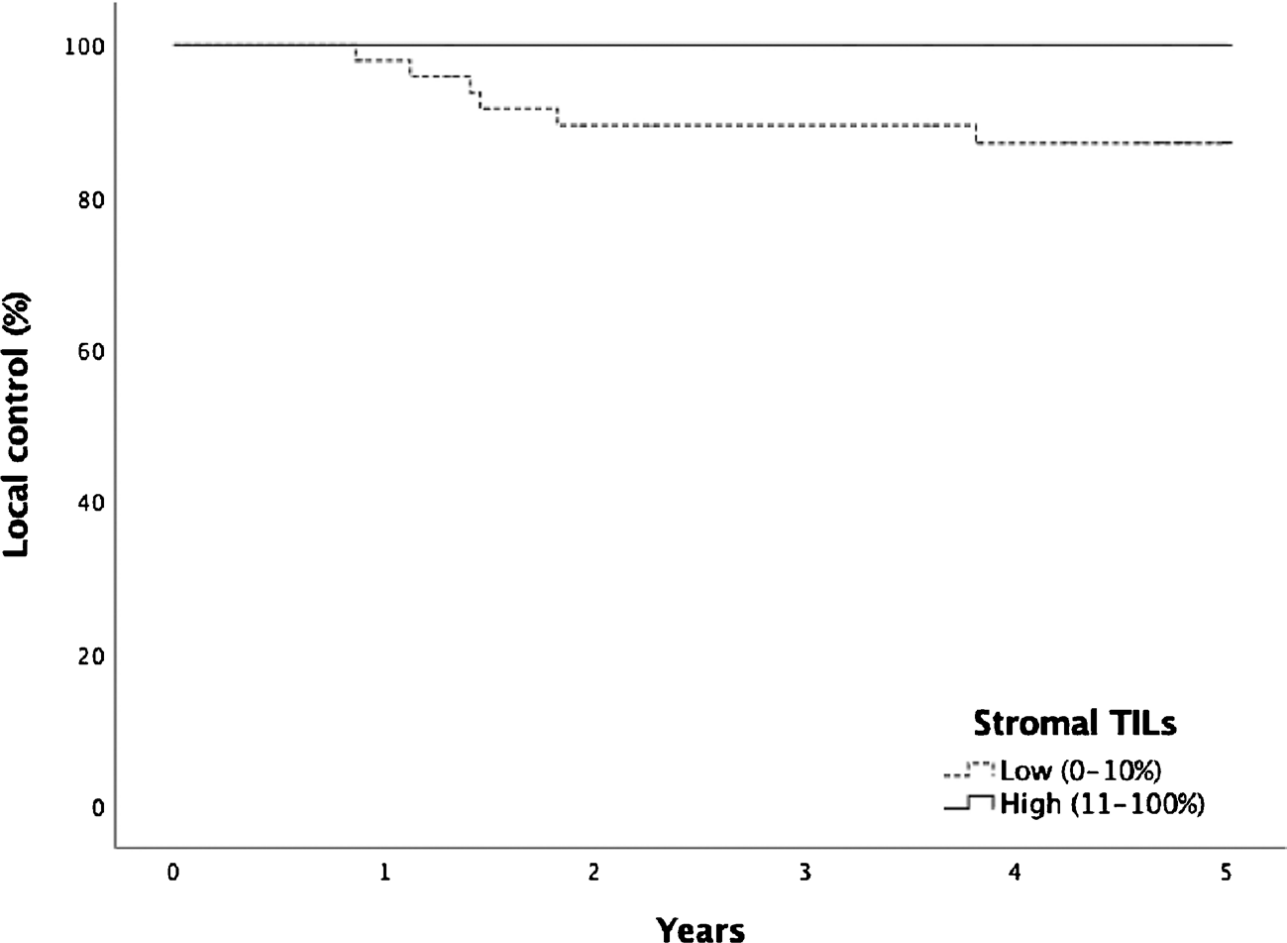
Five-year local control in patients with low and high stromal TIL density (88% vs. 100%, *p* = 0.052)

All 6 of 80 patients with local recurrence had low stromal TIL density. Of 74 patients without local recurrence, 44 had low and 30 high stromal TIL density. TIL percentage was not statistically significantly associated with 5-year local control (*p* = 0.052, Fig. 2). However, low stromal TIL density was significantly more common in patients with either a local recurrence or a new primary tumor of the larynx (detected over 5 years from diagnosis, *n* = 5) compared to patients without any LSCC events during follow-up (low stromal TIL density 91% vs. 58%, *p* = 0.047). Additionally, all 6 patients with local recurrence had low intratumoral TIL density. Of 74 patients without local recurrence, 63 had low and 11 high intratumoral TIL density (*p* = 0.589). Of 6 patients with local recurrence, 5 were treated with surgery and 1 with radiotherapy, but the difference was not statistically significant.

All 3 patients whose TILs were analyzed, and died of LSCC, had low stromal TIL density, but the difference was not statistically significant when compared to LSCC survivors. Larynx preservation rate and second primary tumors were not associated with stromal or intratumoral TIL density.

The patients with both high stromal and tumoral TIL density did not have fewer local recurrences, total laryngectomies or second primary tumors compared to other patients included in TIL analyses. All patients with low stromal TIL density also had low intratumoral TIL density.

### Programmed death-ligand 1 expression and tumor-infiltrating lymphocytes

Combined PD-L1 and stromal TIL analyses included 80 patients. PD-L1 expression was categorized as negative or positive (CPS < 1 vs. 1–100). The patients with high stromal TIL density had higher PD-L1 expression (*p* = 0.037, Table 3). Combined PD-L1 and stromal TILs was not related to sex, smoking, earlier dysplasia, T stage, primary treatment method, larynx preservation rate or second primary tumors. No differences were found in the above-mentioned variables or in 5-year OS, local control, or DSS when the patients with low PD-L1 and stromal TILs were compared to the patients with high PD-L1 and TIL density. Patients with high PD-L1 expression and low stromal TIL density had lower 5-year DSS than other patients (85% vs. 100%, *p* = 0.02), but no differences were found in 5-year OS and local control or other above-mentioned variables.

**Table 3:** The correlation of PD-L1 expression with stromal TILs (*n* = 80)

*N* = 80	PD-L1 < 1 (*n* = 40)	PD-L1 1–100 (*n* = 40)	p value
Low stromal TILs (*n* = 50)	30 (38%)	20 (25%)	0.037
High stromal TILs (*n* = 30)	10 (13%)	20 (25%)	

*PD-L1*, programmed death-ligand 1; *TIL*, tumor-infiltrating lymphocytes

### Laryngeal squamous cell carcinoma deaths

Four patients died of LSCC during a 5-year follow-up and all of them were male smokers. The median age at the time of the LSCC diagnosis was 62 years (range 56–88 years). All patients had T1a tumors affecting the anterior third of the vocal fold. Three patients were primarily treated with surgery and 1 with radiotherapy. LSCC deaths were not associated with sex, age, T stage, or primary treatment method. Of the patients treated with surgery, 2 died after their second recurrence and 1 after his first recurrence. The patient treated with radiotherapy developed a residual tumor after radiotherapy. He had total laryngectomy but died regardless of it. In total 3 patients underwent total laryngectomy. The median time from the primary diagnosis of LSCC to death was 2.4 years (range 1.0–3.9 years).

## Discussion

In our study, the patients with low stromal TIL density more often developed local recurrences or new primary laryngeal cancer (detected more than 5 years from diagnosis) compared to the patients with high stromal TIL density. The cancer cells’ evasion from immunological defense is considered one of the hallmarks of cancer [22]. T cells play an important role in immune response to cancer. According to the protocol, TILs should be analyzed in both intratumoral and stromal areas, and both are associated with the survival of HNSCC [21]. We found no connection between intratumoral TIL density and the prognosis of T1 glottic LSCC. The small size of T1 glottic tumors can affect the behavior of TILs in the tumor microenvironment. However, TILs are a potential histological feature for evaluating T1 LSCC prognosis, and as a method, screening of TILs may be affordable and effortless. T1 LSCC patients with low stromal TIL density might benefit from more intensive follow-up.

Fifty patients had low stromal TIL density and 6 of them (12%) developed a local recurrence. Additionally, 5 of 80 patients analyzed developed a second primary in the larynx and 4 of them had low stromal TIL density. TIL density was not significantly associated with DSS, possibly due to the small number of LSCC deaths, and lack of statistical power. However, all patients who died of LSCC had low stromal TIL density underlining their possible role in controlling tumor spread. In previous studies, low stromal and intratumoral TIL density has predicted shorter survival in HNSCC and LSCC, and especially in recurrent LSCC [7, 15, 16, 19, 20, 23, 24]. In recurrent or persistent LSCC, low TIL density has been associated with lower DSS and RFS [19, 20].

In our study, PD-L1 expression had no prognostic role in T1 glottic LSCC. Of 174 patients, 99 (57%) were PD-L1 positive. PD-L1 negativity could be related to small tumor size and weaker immune response in early-stage laryngeal tumors compared to other solid tumors. In the study by Alessandrini, PD-L1 expression in tumor and immune cells was positive in 15 of 38 (39%) of stage T1-T2 LSCCs and 11 of 32 (34%) of stage T3-T4 LSCCs [9]. The PD-L1’s association with the outcome of HNSCC is contradictory [4–6]. Consequently, PD-L1 can be seen as both a biomarker of constant immune pressure and as an immune response inhibitor. To our knowledge, the prognostic role of PD-L1 in LSCC has been described in 5 studies but those have included both early-stage and advanced-stage LSCC patients. The use of CPS and TPS algorithms varied between the studies [7–9, 25, 26]. Positive association has been found between high PD-L1 and local control, OS, and DSS in stage I-IV LSCC. High PD-L1 expression in tumor cells has been associated with high TIL density in stage I-IV LSCC [7, 8]. Our study confirmed this finding in a series including only T1 glottic LSCC. Additionally, we found that patients with high PD-L1 expression and low stromal TIL density had lower 5-year DSS than had other patients. In such tumor microenvironment, PD-L1 is produced on cancer cells by oncogenic signaling [27]. However, in our series number of patients died of LSCC is small.

We used the PD-L1 clone SP142 instead of clone 22C3 for immunohistochemistry since the former clone was available when our study started. Currently, clone 22C3 is preferred in clinical PD-L1 diagnostics. Clone 22C3 was developed as a selection marker for pembrolizumab and SP142 for atezolizumab [28]. We used slides from one TMA block as control and stained them with both antibody clones and found the PD-L1 expression to be identical. De Ruiter et al. compared different PD-L1 antibody clones in HNSCC and they found significant differences in the percentage of stained cells between the antibodies. However, SP142 was not included in their study [29].

To the best of our knowledge, PD-L1 expression and TILs have not been previously studied and compared in a series of only T1 glottic LSCC. Previous studies have included all stages and subsites of LSCC [7–9]. T1 glottic tumors are small. Therefore, biopsy specimens are not always representative and even clinical diagnostics may be difficult, not to mention immunohistochemistry studies. Accordingly, we used TMA blocks which are not as representative as whole tissue sections. In Finland we have 5 university hospitals and all of them participated in the study. Therefore, our study population is rather comprehensive. All university hospitals run regional biobanks which provided the samples for our study. We included patients treated with both treatment modalities, surgery and radiotherapy, to confirm the correspondence to a real-life setting. The treatment modality was not associated with PD-L1 expression or TIL density.

We studied PD-L1 and TILs as prognostic biomarkers in patients undergoing surgery or radiotherapy, but they also play a role in predicting treatment response to PD-1/PD-L1 inhibitors. Currently, high PD-L1 expression is considered the best predictive biomarker [14]. Therefore, the patients with high TIL density and high PD-L1 expression might be good responders to PD-1/PD-L1 inhibitors. Unfortunately, in our series, all patients who died of metastatic disease had low TIL density. The knowledge concerning LSCC and immune inhibitor response would be relevant since despite the excellent prognosis of T1 glottic tumors 10% of the patients develop recurrent disease and DSS in stage III-IV LSCC remains poor [1].

In conclusion, our results suggest that low stromal TIL density predicts the poorer prognosis of T1 glottic cancer. Moreover, all patients who died of LSCC had low stromal TIL density. Scoring the TIL status at diagnosis would be a relatively easy and inexpensive method for detecting patients who might benefit from more intensive follow-up. The majority of T1 LSCC tumors were positive for PD-L1. PD-L1 expression was not associated with the prognosis of T1 glottic LSCC. Accordingly, T1 glottic LSCC tumors with high stromal TIL density had high PD-L1 expression, which may reflect their parallel role in immune activation in T1 glottic LSCC.

## Data Availability

The datasets generated during and/or analyzed during the current study are available from the corresponding author on reasonable request.
