# Correction to: Programmed death-ligand 1 and tumor-infiltrating lymphocytes (TILs) – low TIL density may predict poorer long-term prognosis in T1 laryngeal cancer

**DOI:** 10.1007/s00428-023-03618-2

**Published:** 2023-08-09

**Authors:** Pihla Pakkanen, Taru Ilmarinen, Elina Halme, Heikki Irjala, Petri Koivunen, Matti Pukkila, Sami Ventelä, Alhadi Almangush, Eva-Maria Birkman, Outi Lindgren, Virva Pohjolainen, Nelli Sjöblom, Caj Haglund, Jaana Hagström, Leena-Maija Aaltonen

**Affiliations:** 1 Department of Otorhinolaryngology - Head and Neck Surgery, University of Helsinki and Helsinki University Hospital, Helsinki, Finland; 2 Department of Otorhinolaryngology - Head and Neck Surgery, University of Tampere and Tampere University Hospital, Tampere, Finland; 3 Department of Otorhinolaryngology - Head and Neck Surgery, University of Turku and Turku University Hospital, Turku, Finland; 4 Department of Otorhinolaryngology - Head and Neck Surgery, University of Oulu and Oulu University Hospital, Oulu, Finland; 5 Department of Otorhinolaryngology - Head and Neck Surgery, University of Eastern Finland and Kuopio University Hospital, Kuopio, Finland; 6 Department of Pathology, University of Helsinki and Helsinki University Hospital, Helsinki, Finland; 7 Department of Pathology, University of Turku and Turku University Hospital, Turku, Finland; 8 Department of Pathology, University of Oulu and Oulu University Hospital, Oulu, Finland; 9 Fimlab Laboratories, Department of Pathology, Tampere University Hospital, Tampere, Finland; 10 Department of Surgery, University of Helsinki and Helsinki University Hospital, Helsinki, Finland; 11 Department of Oral Pathology and Radiology, University of Turku, Turku, Finland

**Correction to: Virchows Archiv**

**https://doi.org/10.1007/s00428-023-03586-7**

The Tables of original published version of the above article were incorrect. Tables 1 and 2 and its citation should have been Tables 2 and 3. The correct tables are shown as follows:

**Table 1:** Clinical characteristics, and expression of PD-L1 and TILs in patients with T1 glottic LSCC (N=174)

Characteristic	All patients (N=174), n (%)	PD-L1 (n=174), n (negative, low, high)	Stromal TILs (n=80), n (low, high)
Sex			
Male	146 (84)	146 (64, 56, 26)	70 (45, 25)
Female	28 (16)	28 (11, 11, 6)	10 (5, 5)
Smoking history			
Yes	147 (84)	147 (58, 59, 30)	70 (43, 27)
No	19 (11)	19 (12, 6, 1)	7 (6, 1)
NA	8 (5)	8 (5, 2, 1)	3 (1, 2)
Earlier dysplasia*			
Yes	12 (7)	12 (5, 5, 2)	6 (4, 2)
No	143 (82)	143 (62, 55, 26)	68 (44, 24)
NA	19 (11)	19 (8, 7, 4)	6 (2, 4)
Histological grade			
Grade 1	50 (29)	50 (23, 15, 12)	14 (11, 3)
Grade 2	51 (29)	51 (21, 24, 6)	23 (16, 7)
Grade 3	5 (3)	5 (2, 2, 1)	3 (2, 1)
NA	68 (40)	68 (29, 26, 13)	40 (21, 19)
T stage			
T1a	156 (90)	156 (64, 62, 30)	73 (45, 28)
T1b	18 (10)	18 (11, 5, 2)	7 (5, 2)
Primary treatment			
Surgery	100 (57)	100 (45, 34, 21)	53 (30, 23)
Radiotherapy	74 (43)	74 (30, 33, 11)	27 (20, 7)
Local recurrence			
Yes	20 (11)	20 (8, 7, 5)	6 (6, 0)
No	154 (89)	154 (67, 60, 27)	74 (44, 30)
Died of LSCC			
Yes	4 (2)	4 (0, 3, 1)	3 (3, 0)
No	170 (98)	170 (75, 64, 31)	77 (47, 30)

Abbreviations: LSCC, laryngeal squamous cell carcinoma; NA, not available; PD-L1, programmed death-ligand 1; TIL, tumor-infiltrating lymphocyte

*presence of dysplasia confirmed in laryngeal biopsy specimens before the diagnosis of T1 glottic cancer

**Table 2:** The correlation of combined positive score (CPS) and tumor proportion score (TPS) in T1 glottic cancer

N=174	TPS negative (n=92)	TPS low (n=66)	TPS high (n=16)
CPS negative (n=75)	68	7	0
CPS low (n=67)	23	41	3
CPS high (n=32)	1	18	13

Abbreviations: CPS, combined positive score; TPS, tumor proportion score

**Table 3:** The correlation of PD-L1 expression with stromal TILs (n=80)

N=80	PD-L1 < 1 (n=40)	PD-L1 1-100 (n=40)	p value
Low stromal TILs (n=50)	30 (38%)	20 (25%)	0.037
High stromal TILs (n=30)	10 (13%)	20 (25%)	

Abbreviations: PD-L1, programmed death-ligand 1; TIL, tumor-infiltrating lymphocytes

The original article has been corrected.

